# Nursing Management and Adverse Events in Thyroid Cancer Treatments with Tyrosine Kinase Inhibitors. A Narrative Review

**DOI:** 10.3390/cancers13235961

**Published:** 2021-11-26

**Authors:** Aurora De Leo, Emanuele Di Simone, Alessandro Spano, Giulia Puliani, Fabrizio Petrone

**Affiliations:** 1 Nursing: Technical, Rehabilitation, Assistance and Research Department-IRCCS Istituti Fisioterapici Ospitalieri and Regina Elena National Cancer Institute, 00144 Rome, Italy; aurora.deleo@ifo.gov.it (A.D.L.); emanuele.disimone@ifo.gov.it (E.D.S.); fabrizio.petrone@ifo.gov.it (F.P.); 2 Biomedicine and Prevention Department, Tor Vergata University, 00133 Rome, Italy; 3 Oncological Endocrinology Unit, IRCCS Regina Elena National Cancer Institute, 00144 Rome, Italy; giulia.puliani@ifo.gov.it; 4 Department of Experimental Medicine, Sapienza University of Rome, 00185 Rome, Italy

**Keywords:** thyroid cancer, tyrosine kinase inhibitors, multikinase inhibitors, adverse events nursing management, nursing care

## Abstract

**Simple Summary:**

Tyrosine kinase inhibitors are an effective and promising therapy in the treatment of advanced differentiated medullary thyroid cancers. The prevention and management of new adverse events of these drugs are important to keep patients on their treatment course, avoiding drug discontinuation or interruption, and are associated with a faster recovery of the disease. The contribution of a multidisciplinary team of healthcare professionals optimizes the management of adverse events, maximizing the benefits and reducing the risks of treatment, consequently improving the quality of life of patients.

**Abstract:**

Background: The advent of multikinase inhibitors has changed the treatment of advanced, metastatic, unresectable thyroid cancers, refractory to available treatments. These drugs cause new adverse events that should be prevented and treated for long periods, and sometimes beyond their discontinuation. The purpose of this narrative review was the description, prevention, and nursing management of the most frequent adverse events of locally advanced or metastatic differentiated thyroid cancer with sorafenib and lenvatinib, and medullary Thyroid cancer with vandetanib and cabozantinib treatment. Methods: A narrative literature review. Results: Studies included in this narrative review suggest that over 90% of patients treated with tyrosine kinase inhibitors experience at least 1 adverse event of any grade affecting their quality of life. Patients treated with tyrosine kinase inhibitors experienced at least one adverse event at any grade in ≥90% of cases, with a higher incidence in the first 6–8 weeks of treatment. The most frequent adverse events that can affect a patients’ quality of life are dermatological, gastrointestinal, cardiovascular, and metabolic. Conclusions: Early assessment of risk factors and identification of adverse events can help nurses support these patients throughout their clinical-therapeutic pathway, increasing the benefits of treatment and reducing reduction/discontinuation.

## 1. Introduction

The 21st century has seen the birth and development of immunotherapy and molecularly targeted therapy (MKI), which have supported treatments for thyroid cancer responsive to tyrosine kinase inhibitors (TKIs). This innovative therapeutic approach brought changes to therapeutic settings: hospital treatment cycles are often replaced by medication administered at home, for months, years, and sometimes a lifetime. In regards to traditional cytotoxic chemotherapy, the most frequent adverse events (AEs) consist of mainly nausea, alopecia, and mucositis [1] which have to be managed with symptomatic therapy, for a time often limited to cycles of planned treatment. Alternatively, adverse effects of new drugs can affect the lives of patients for long periods of time as well as change over time, sometimes even beyond their interruption, having a meaningful impact on quality of life (QoL) [2]. According to Roset et al. [3], toxicities experienced by patients treated with TKIs have a greater impact on health-related quality of life (HRQoL) than non-targeted therapies. The precise and long-lasting detection of new AEs increasingly managed by patients at home, far from hospital/outpatient settings, appears to be of considerable importance. Furthermore, the difficulty of monitoring the most important self-reported AEs and patient-reported outcomes (PROs) [4] is well known, and should be taken into consideration in clinical practice. Evaluation of treatment-related toxicities usually focuses on the assessments carried out by clinicians within clinical trials, and collected through clinical tools, such as the common terminology criteria for AEs (CTCAE) ver. 5.0 [5]. The CTCAE is a tool for evaluating the signs, symptoms, and diseases arising during treatment, even if unrelated to the therapy. There are 5 grades of severity of AEs: 1 (mild), 2 (moderate), 3 (severe), 4 (dangerous for patient’s life), and 5, with the AE-related death of patients. As reported by Laugsand et al. [6], many of the AEs patients experience are either missed or underestimated by clinicians, consequently having a negative impact on the management and control of symptoms, therapeutic relationship, patient satisfaction and QoL.

### 1.1. Thyroid Cancer

Thyroid cancer is the most common endocrine neoplasm [7,8]. It accounts for 1% [7] to 3% [8] of all new cancer cases, with an incidence of 15.7 cases per 100,000 persons [8].

There are three major groups of thyroid cancer [9]: differentiated thyroid cancers (DTCs), medullary thyroid cancers (MTCs), and anaplastic thyroid cancers (ATC), respectively 93–95%, 5%, and 1–2% of all thyroid cancers.

Differentiated forms have an excellent prognosis in about 90% of cases [10], due to radical surgery gland resection with loco-regional lymphadenectomy being performed if necessary. Thyroid-stimulating hormone (TSH) suppression and adjuvant therapy with radioiodine ablation are additional treatments for only a few selected patients [11]. However, approximately 4–13% of patients have advanced, metastatic, poorly differentiated or undifferentiated forms of radioactive iodine (RAI) refractory cancer [12]. The post-surgical follow-up involves the assay of a specific biomarker of DTC, serum thyroglobulin (Tg), which is detected for prognostic purposes together with anti-thyroglobulin auto-antibodies (TgAb) [11]. The presence of TgAb, which should be absent in patients after total thyroidectomy, limits the reliability of the specific biomarker in about 30% of cases [13].

MTCs are neuroendocrine RAI refractory thyroid cancers [14] that can either be sporadic or familial. The two specific tumor markers are calcitonin (CTN) and carcinoembryonic antigen (CEA). Familial forms can develop into thyroid cancer alone or can associate thyroid cancer with other cancers, resulting in multiple endocrine neoplasia (MEN) [7]. The sporadic forms involve 75% of cases and have a similar prognosis to DTCs [2]. Hereditary MTC is caused by an autosomal dominant mutation in 25% of the cases [15] of the RET proto-oncogene related to more aggressive forms [16], giving rise to multiple endocrine syndromes (multiple endocrine neoplasia MEN1, MEN2 MEN3 and the recently discovered MEN4) [17]. MEN are genetic autosomal dominant syndromes which are characterized by genetic abnormalities, affecting endocrine glands and presenting clinical signs. Each type is characterized by a different association of pituitary, pancreatic, parathyroid, medullary thyroid, renal, reproductive and adrenal cancers [17]. The presence of MTC and metastatic lymph node involvement is usually asymptomatic for a long period of time, may cause dysphagia and/or dysphonia, respectively, and may cause difficulty in swallowing and speaking.

A completely different type of medical history concerning anaplastic thyroid cancer (ATC), known as the most undifferentiated form, is lethal for patients in the first 1–6 months from diagnosis [2]. For patients with ATC, the guidelines of the American Thyroid Association indicate that surgery very rarely resolves the issue, delaying treatment with radiotherapy and chemotherapy for palliative purposes, and often resorting to a rescue tracheostomy [11].

Scientific advances and deeper knowledge of the human genome have generated significant results in the treatment of thyroid cancers, leading to the development of MKIs, which have changed the available treatments for unresectable, advanced, metastatic, refractory thyroid cancer. Although less so than cytotoxic chemotherapy, these treatments also affect patient QoL [18,19], including their physical, psychological, sexual, social, relational, affective, cognitive, economic and perceptive well-being. 

Excluding ATC, thyroid cancers generally have a favorable prognosis, with indolent growth and mainly only loco-regional metastasis. Surgical treatment is often curative [11,14], while, in advanced and metastatic forms, the following treatments are used TKIs, and best supportive care (BSC), supportive palliative therapy for symptom and pain control with the use of radiotherapy, palliative surgery, symptomatic therapy and pain therapy [11,20].

### 1.2. Tyrosine Kinase Inhibitors

The most widely used MKIs in Italian clinical practice for the treatment of advanced thyroid tumors which have been approved by the Food and Drug Administration and the European Medicines Agency are [21,22,23,24]: vandetanib and cabozantinib, approved for the advanced MTC, and sorafenib and lenvatinib approved for advanced DTC. For patients with both locally advanced or metastatic DTC and MTC, TKIs represent an effective and cytostatically promising therapy which counteracts cancer proliferation and new angiogenesis [25]. The therapeutic choices and the mechanisms of action of the different drugs [2,10,21,22,23,24,26,27] will not be the subject of this paper, but will be briefly described within the context of the management and prevention of the most common AEs in TKIs.

In agreement with the 2015 American Thyroid Association Guidelines [11,14], thyroid cancer patients eligible for the use of TKIs are affected by unresectable metastatic forms, with multiple and diffused lesions, as shown in radiological progression according to RECIST criteria [28], when compared to the previous 6–12 months. As has been observed by preliminary studies [21,22,23,24], TKIs produce AEs that would be effectively prevented, detected, and managed, to preserve patient QoL. The prevention, early detection and management of the new AEs of these drugs represent a strategic point for physicians and nurses to keep patients on their treatment course, often *nam omne vitae*, maximizing the benefits and reducing the risks of these treatments.

The purpose of this narrative review was the description, prevention and nursing management of the most frequently occurring adverse events in patients with locally advanced or metastatic differentiated thyroid cancer treated with sorafenib and lenvatinib, and patients with medullary thyroid cancer undergoing vandetanib and cabozantinib treatment.

## 2. Materials and Methods

**Figure 1:**
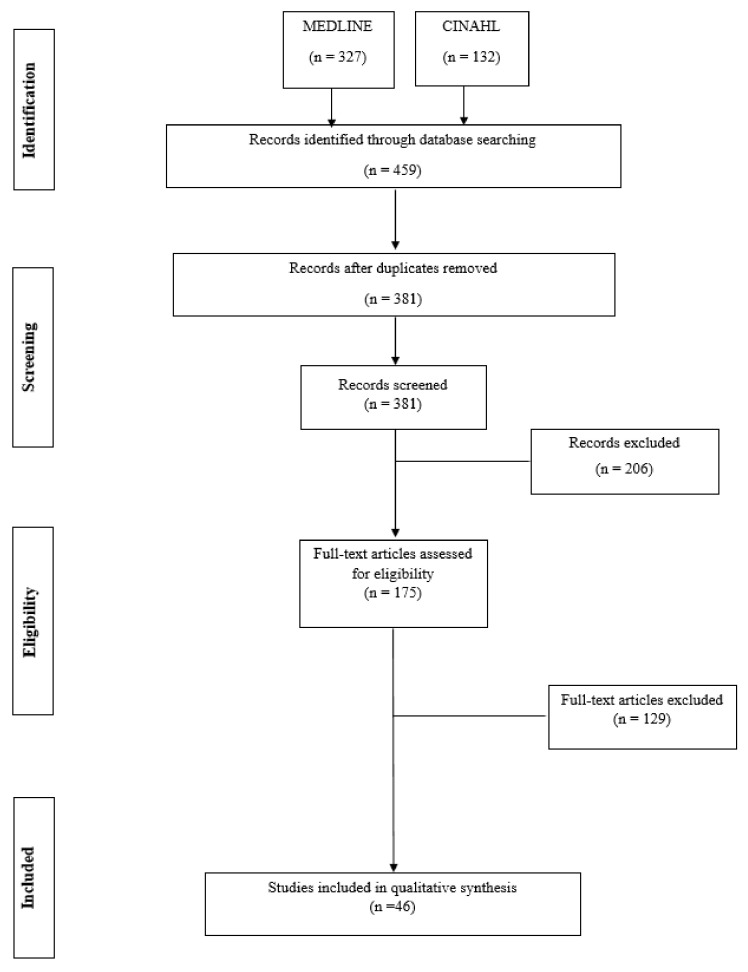
Screening process.

A brief narrative literature review was conducted. A search on MEDLINE and CINAHL was carried out from September 2020 to January 2021, limited to articles written in English from 2000 to 2020, with the following terms in the title: tyrosine kinase inhibitors, multikinase inhibitors, sorafenib, lenvatinib, cabozantinib and vandetanib, combined with (AND) thyroid cancer. Both quantitative and qualitative studies were included. The MEDLINE database produced 327 studies and 132 from the CINAHL database. Duplicates were excluded and led to 381 studies that were read in title and abstract. 206 studies were excluded as irrelevant, and the remaining 175 were read in full text. The screening process led to the selection of 46 studies included in the review (Figure 1). RefWorks^®^ software was used to select the studies.

All studies that analyzed the use of chemotherapeutic agents with a focus on adverse events and their nursing management were selected. In addition, all studies that had to consider QoL as an outcome were also selected.

## 3. Results

Of the 459 results, 46 articles were deemed relevant (Figure 1).

Four important phase III clinical trials led to the approval of these four drugs [21,22,23,24]. The most common side effects of these oral administrations shown in Table 1 are similar and generally moderate in grade dermatological, gastrointestinal, cardiovascular and metabolic disturbances [21,22,23,24]. The frequency of AEs is higher in the first 6–8 weeks of treatment [21,22,23,24], so initially, clinical surveillance necessitates even closer vigilance.

AEs can be monitored by the clinicians in accordance with the common terminology criteria for AEs (CTCAE) ver. 5.0 [5]. These mechanism-based toxicities attributable to the specific agents that are inhibited by the drug can be proactively prevented and counteracted in terms of their high frequency and impact on patient QoL. Similar toxicities in different treatments can be prevented and addressed, as further described, after a brief description of the four TKIs. Typically, almost all patients on treatment experience at least one grade 1–2 AE and, depending on the drug and the individual responses to the drug, toxicities can also be severe [21,22,23,24]. Grade 1–2 AEs are usually managed with medication and sometimes with dose reduction, while grade 3–4 toxicities may not respond to medication and require drug discontinuation, then resumption at low doses after the resolution of the AEs [29].

The results were categorized according to specific categories of interest, as shown below.

### 3.1. Vandetanib

Vandetanib is an oral bioavailable TKI suitable for adults and children over 5 years of age who are affected with unresectable and metastatic MTC, and are effective in hereditary and sporadic forms [24,30]. The coated tablets are available at doses of 100 and 300 mg. The recommended dose of vandetanib is 300 mg to be taken at the same time each day, whether on a full or empty stomach [31] and its half-life is 19 days, a period of time to consider in the management of the AEs. Typically, almost all patients on treatment, experience at least one grade 1–2 AE, where 55% of them have at least one severe grade AE [24,32]. A total of 8% of patients experienced QT prolongation, which can be a life-threatening adverse event, is dangerous especially for torsades de pointes. Cardiovascular toxicities, especially hypertension, have been reported in up to about 25% of cases [33], therefore in congenital or more severe cases of heart disease, vandetanib may be contraindicated. Other common side effects regarding the use of vandetanib include electrolyte and metabolic disturbances (particularly glycidic and hepatic), fatigue, headache, hypothyroidism and adrenal hypo [31]; less frequent AEs involve intestinal hemorrhages and gastrointestinal and tracheal fistulas [34]. Careful monitoring of heart disorders and blood pressure is particularly indicated with the use of vandetanib.

There is a lack of data regarding the efficacy of using the two markers CT and CEA for monitoring MDC progression with vandetanib and their prognostic value [35].

### 3.2. Cabozantinib

Cabozantinib is the second TKI approved for the treatment of advanced MTCs [21]. Cabozantinib is available in oral formulation, at a dose of 20, 40 and 60 mg, containing lactose, which is to be taken in a single fasted state at least 2 h before a meal and at least 1 h after taking cabozantinib. The initial and full dose of cabozantinib is 140 mg, and tablets should be taken whole and not crushed [7]. Grade 3 or 4 AEs were reported in 69% of patients, including diarrhea, palmar-plantar erythrodysesthesia (or hand-foot skin reactions, HFSR), fatigue and hypertension. Severe gastrointestinal and non-gastrointestinal bleeding, perforation and fistulae may occur in approximately 10% of patients, which can be possibly life-threatening. Hypothyroidism and electrolyte imbalances are the most frequent metabolic alterations, occurring in more than 50% of patients. About 80% of patients experience at least a reduction in therapy and about 50% discontinuation due to severe toxicities [21]. Grade 5 AEs, in which death is related to the severity of the AEs, can occur in 5–10% of patients due to fistulas, respiratory and multi-organ failure, cardiotoxicity, hemorrhages, sepsis and cachexia.

### 3.3. Sorafenib

Sorafenib is a TKI approved for the treatment of patients with advanced RAI-refractory DTCs [22]. It is an oral TKI in 200 mg coated tablets, active at the recommended dose of 400 mg twice a day, for a total daily dose of 800 mg. Grade 3–4 AEs with sorafenib usually occur in 35% [22] to 45% of cases [36] in the first 3 months of treatment and include HFSR, hypertension, diarrhea, fatigue, weight loss, rush and dyspnea. Hypothyroidism, hypocalcemia and electrolyte imbalances are generally common, while grade 5 toxicities are rare and are caused by myocardial infarction [22].

### 3.4. Lenvatinib

Lenvatinib is the last oral TKI used in the treatment of RAI-refractory DTCs [23]. The hard capsules should be taken every day at the same time with water, and should swallowed whole either on an empty or full stomach. In cases of dysphagia in patients with advanced disease, the capsules can be dissolved in a spoonful of water or apple juice for at least 10 min. The recommended daily dose is 24 mg with two 10 mg capsules and one 4 mg capsule. The incidence of AEs is about 75% of cases. The most frequent AEs with Lenvatinib are hypertension, diarrhea, fatigue, anorexia, weight loss, nausea, stomatitis, HFSR and proteinuria. In particular, any-grade hypertension is a very common AE, grade ≥ 3 in more than 40% of cases, therefore close monitoring of blood pressure on a daily basis is required in these patients. Increases in circulating TSH levels and proteinuria are also common, respectively in 60% and 30% of cases [23]. QT prolongation, arterial and venous thromboembolic effects, renal and hepatic failure, gastrointestinal fistula and posterior reversible encephalopathy syndrome can occur in 5% to 8% of patients. A recent study [37] suggests that reducing the dose of lenvatinib due to related toxicities does not decrease the efficacy of the treatment, and patients are kept on therapy longer with better outcomes.

### 3.5. AEs’ Nursing Management

Patients treated with TKIs have to continue therapy until disease progression or unacceptable/life-threatening toxicities, refractory to dose reduction or medical therapy [2]. Therefore, the prevention and timely management of TKI-related AEs are strategic points in these long therapeutic pathways to keep patients on therapy for as long as possible. The mechanism of onset of any-grade toxicities is unclear, and may probably be due to a set of cofactors, including genetic and environmental ones, however, higher-grade toxicities are dose-related [21,22,23,24]. As was observed by Henry et al. [38], even thyroid cancer patients have a generally favorable prognosis in regards to ng prognosis and symptom management of their disease and benefit from adopting a multidisciplinary approach. Even in thyroid cancer patients, more information is associated with a better perception of the disease, and with less distress and improved QoL [39]. In evaluating the impact of advanced thyroid cancer treatments on QoL, the SF-6D (short-form six-dimension) and EQ5D instruments can support patient clinical pathways [40] in addition to the CTCAE [4]. The EuroQoL (EQ5D) [41] is a simple self-assessment tool used to evaluate 5 dimensions of health-related (mobility, self-care, habitual activities, pain/discomfort, anxiety/depression). The SF-6D [42] is the shortened version of the SF36, which more rapidly investigates the self-perception of six dimensions (physical functioning, role limitations, social functioning, pain, mental health and vitality). The use of the distress thermometer has also been recommended [43]. It is a simple and handy tool recommended for screening discomfort in cancer patients by the National Comprehensive Cancer Network (NCCN). With this simple tool, patients indicate their degree of distress on a scale of 1 (no distress) to 10 (extreme distress), and they identify the source of their distress from the problem list provided. The management of AEs depends on the level of severity [4]: a dose reduction may be sufficient for mild toxicity (≤1), while for grade 2 and 3 toxicities, medical therapy and interruption of treatment may be necessary, even permanently so in case of grade 4 toxicity. The contribution of a multidisciplinary healthcare professional team (HCPs) is recommended in the management of TKIs [44], as is the support of a caregiver in the early stages of the therapeutic pathway [45].

EKG, cardiological examination and blood tests should be carried out before starting treatment with TKIs, and then repeated at baseline and after 2–4–8–12 weeks, possibly with performing an endocrinological or oncological examination every 15 days in the first two months of treatment [10]. Subsequently, in the absence of complications, the follow-up can be extended even every 1–3 months. In elective surgeries, discontinuation of TKIs before and after surgery should always be taken in to account. Generally, TKIs should be discontinued between 2–4 weeks before major surgery, or 10 days before any surgery and can be resumed at wound healing [46,47].

Dermatological AEs are common in these patients, including skin rash, hyperkeratosis, photosensitivity, damage to nails and hair, itching, mucositis and bruises, but they are usually not life-threatening. Some lifestyle changes are necessary, such as healthy diet, moderate daily physical activity, careful body and oral hygiene, use of emollient creams and neutral soap, avoid exposure to too high and too low temperatures, limit sun exposure, use of cotton gloves in household chores, maintain a good sleep-wake rhythm. Patient diaries are a useful way to note the most common AEs, suggested and applied remedies, therapeutic dosage and any variations and observations that have occurred. Patient self-reporting is also valuable tool for the care team to monitor the patient’s perceived health status, and compare it to the health status of an objective measuring tool such as CTCAE [4].

TKIs should be taken every day at the same time and dosage, with variations in dose to be agreed with the clinician or an expert nurse. Skipping doses can be taken no less than 12 h after the next dose, never should two doses be taken at the same time. The effectiveness of the drug is not directly proportional to the AEs produced, but in general, the AEs are dose-related [21,22,23,24] and their incidence is higher in the first 2 months of treatment. They usually disappear with drug discontinuation, but sometimes they could continue even after. Moreover, TKI interactions with other drugs may impair their absorption. Before starting treatment, a basic physical examination is very important to remove all possible causes of future interruptions of therapy, such as skin and mouth lesions and hypertension. Nurses should perform a thorough body check at each patient visit to assess any possible grade of toxicity. Having a telephone number to call for any patient needs will reduce the feeling of loneliness that cancer patients may experience at different stages of the disease [48].

### 3.6. Proactive Counseling

The choice of the drug has to be tailored to maximize the benefits, consequently reducing risks and discomfort for patients [49]. All worsening symptoms triggered by the drug should be evaluated and possibly be removed at the baseline. These include oral cavity, feet, nails, skin, calluses, hypertension, metabolic alterations, nutrition, physical activity, psycho-social disorders, anxiety and depression. Patients should be referred to the care of specialists to promote lifestyle modifications and remove lesions or conditions that have been worsened by therapy, require reduction in doses or treatment interruptions. Hyperglycemia and hypoglycemia may occur during therapy with TKIs, because of drug interference with the glucose metabolism [50]. It is important for patients to keep a daily diary recording blood glucose levels detected on an empty stomach and two hours after meals; do moderate-to-steady physical exercise such as a 30-min walk daily and keep a balanced diet. Mild hyperglycemia benefits from diet and exercise, while high values require endocrinological management [51]. Through specialized counseling, patients should be informed of the 90% to 100% chance of experiencing at least one any-grade AE [21,22,23,24], during their therapeutic pathway. Good prevention and management will allow patients to reduce the impact on their QoL, including the completion of a daily diary to assess the onset and extent of major AEs experienced.

### 3.7. Practical Advice

#### 3.7.1. Palmar Plantar Erythrodysesthesia or Hand-Foot Skin Reactions (HFSR)

These disabling skin lesions can worsen with trauma from pressure, friction, exposure to excessively hot or cold temperatures, or exposure to radiation, including sun radiation. In the case of HFSR, the skin is blistered, thickened and callused on the palms and soles of the hands and feet, as well as erythema and skin rash.

Prevention: remove hyperkeratotic areas with calluses and thickening from hands and feet before starting treatment according to the patient’s overall health status. Use 10% urea-based creams up to 2–3 times a day on hands and feet, wear gloves and cotton socks, avoid high-heeled and tight shoes, wear comfortable shoes, avoid washing with hot or cold water, dry hands and feet well, especially in the inter-digital spaces, use alcohol-free moisturizers, detergents and sunscreen (SPF ≥ 30). Take care of the skin of the hands and feet, in particular avoiding long and tiring walks and lifting excessive weights with hands.

AE practical advice includes use of creams with 20–40% urea on affected areas of the skin such as flaking, and specific products on the callused areas. Baths with warm water with magnesium sulfate can relieve pain. In case of grade 2/3 toxicity, topical or systemic steroids, antibiotics and analgesics can be prescribed. Carry out periodic checks with a podiatrist.

#### 3.7.2. Skin Rash

Rashes significantly affect patient QoL and their impact can be detected with appropriate tools, such as the dermatology life quality index (DLQI) [52], a 10-item questionnaire to investigate the impact on QoL of dermatological lesions. Each question can be assigned a score from 0 to 3, where 0 indicates no impact on QoL and 3 indicates extreme impact (total score 0–30). Topical or oral antibiotics and corticosteroid-based compounds are used to treat skin rashes.

Prevention methods include alcohol-free emollient creams and soaps, avoiding hot or cold water, and avoiding direct and prolonged exposure to sunlight. Use comfortable clothes made from natural fibers and avoid drying by rubbing with frictional force but by patting the skin instead. The use of sunscreens, hats and protective clothing is recommended to reduce the incidence of dermatological toxicities associated with worsening QoL, especially in young patients [53].

AE practical advice indicates the reaction is generally mild to moderate, but in the event of severe rash, a dermatologist may prescribe topical or systemic steroids.

#### 3.7.3. Stomatitis and Mucositis

This condition is very disabling for patients who may have difficulties with pain in feeding and speaking. It is a painful inflammation that involves the mouth (stomatitis) and sometimes the digestive system (mucositis). Owing to its consequences, this disorder is more functional than anatomical, and it should be treated with adequate nutritional support and symptomatic therapy for pain control [54]. Pre-treatment and periodic scaling of tartar are recommended. Periodical evaluations of the patient’s nutritional status and support from a dietician are recommended. Pre-treatment and intermittent visits to the dentist are recommended.

Prevention: avoid irritating foods (spicy, salty, sour or hot), preference of soft foods, good oral hygiene (soft toothbrush and fluoride toothpaste before bedtime, rinses with alcohol-free mouthwashes), moisturize the lips with emollient balm, and use antifungal preparations as needed. Rinsing with a cold solution of water, chamomile, and one teaspoon of baking soda can relieve the mouth after meals.

AE practical advice: observe strict oral hygiene after every meal, use medicated mouthwashes and avoid commercial alcohol-based toothpaste and peppermint.

#### 3.7.4. Diarrhea

Diarrhea is a common treatment-related AE associated with the use of TKIs. In the case of diarrhea, it is important to evaluate its duration, type and exclude any secondary causes (bacterial or viral infections, wrong diet). Increased calcitonin in MTC and TKIs can induce diarrhea, which can cause dehydration and electrolyte disorders [26]. Patient education should include a symptom diary recording the number and severity of episodes, foods eaten and related symptoms.

Prevention: limit the intake of milk and dairy products (preferring lactose-free foods), dried fruit, figs, grapes, foods rich in fibers, and legumes. Drink at least two liters of water a day and keep a daily diary for the income-expenditure balance.

Suggested foods: high-protein and low fiber foods, fish, low-fat meat, eggs, poultry, highly digestible milk without lactose, yoghurt, lean cheese, cooked vegetables (carrots and peas), freshly peeled fruit, and lactose-free products.

Foods to avoid/limit: fatty or fried foods, whole milk, alcohol, tea, coffee, fatty cheese, raw vegetables with thick skins and seeds, or very fibrous, nuts, chewing gum and candy.

AE practical advice: small, light and frequent meals, hydration (2 L/day), periodically check weight and electrolytes, constantly monitor the number and quantity of discharges, take probiotics and loperamide as needed starting at doses as low as 4 mg then up to 2 mg every 4 h, or after each bowel movement, in order to avoid dehydration. Daily monitoring of the physiological energy expenditure and balance.

#### 3.7.5. Hypertension and QT Prolongation

In general, hypertension and cardiovascular toxicities can be life-threatening AEs, especially in patients receiving vandetanib [24]. Before starting treatment, all patients should control their blood pressure. Risk factors potentially associated with hypertension, such as obesity, family history and smoking should be evaluated. Blood pressure should be kept below 140/90 mm/Hg [55] and checked daily, even several times a day in the first 2 months of treatment [31]. Under vandetanib therapy, EKG cardiac monitoring should be done every 1–2 weeks in the first month of treatment, then once a month for the first three months, and then according to the patient’s overall health status [31]. The importance of nursing education on performing and interpreting a 12-lead EKG electrocardiogram to avoid diagnostic delays, misinterpretation and misdiagnosis are well known [56]. Electrolytes must also be monitored.

Prevention: EKG, cardiological examination, close daily monitoring of blood pressure especially in the first 2–3 months of treatment, healthy diet and regular physical activity, reduce the consumption of salt, alcohol, smoking, tea and coffee, and reduce stress. Multiple TKI interactions with many antihypertensive drugs [57,58] have to be considered.

AE practical advice: close daily blood pressure monitoring and timely prescription of antihypertensive agents if necessary. Close hydro-electrolyte monitoring during the use of diuretics, because of the frequent electrolyte imbalances, to avoid dehydration and cardiovascular imbalance, especially in case of vomiting and diarrhea [57,58].

#### 3.7.6. Fatigue

Fatigue is a very common AE in patients receiving TKIs [21,22,23,24] due to both the psychological and physical mechanisms involved. An assessment of the severity of fatigue can be performed with the brief fatigue inventory (BFI), a simple and practical tool that detects the severity of the patient’s fatigue [59], alongside the SF-6D instrument for QoL assessment [40]. The BFI six interference items (general activity, mood, walking ability, normal work including housework, relations with other people, enjoyment of life) are associated with standard quality-of-life measures (fatigue right now, usual fatigue in last 24 h, worst fatigue in last 24 h) to assess cancer-related fatigue. It is important to make a differential diagnosis with confounding factors leading to fatigue, such as diarrhea, anemia, hypothyroidism, low testosterone levels and depression. In the treatment of hypothyroidism, the management of levothyroxine is important. Both generic formulations and the brand-name of levothyroxine have shown the same efficacy in stabilizing thyrotropin levels in thyroid hormone replacement therapy [60]. On the other hand, it is important to know that the American Thyroid Association [61] recommends avoiding switching from branded to generic drug formulations while on therapy. Furthermore, to allow optimal absorption of the drug, it is recommended to take it in a fasted state 60 min before breakfast or 3 h after one of the main meals of the day. It is also recommended to avoid other medications when taking levothyroxine, in impaired absorption (for example with gastric protectors composed of calcium and iron), postponing them after 2–4 h. Gastrointestinal disorders have to be considered in absorption. Any dose adjustments of levothyroxine should be considered in case of large variations in weight, pregnancy status and age, while taking into account the half-life of the drug of approximately 6 weeks. The state of mild hypothyroidism generally causes fatigue and transient water retention, while iatrogenic hyperthyroidism can lead to numerous side effects, including fibrillation and osteoporosis. Finally, levothyroxine serum levels should be collected without taking the drug in the morning of the blood sample [61].

Prevention: Consider small rest periods during the day, engage in moderate exercise and practice a balanced diet, healthy lifestyle, reduce stress and stressful activities, preserve a good sleep/wake rhythm, support the patient in identifying achievable goals [62]. In order to reduce the fatigue experienced during the day it may be helpful to take TKIs in the evening instead of in the morning [46]. In patients being treated with lenvatinib, exercise is not recommended as it may worsen fatigue [63].

AE practical advice: evaluate the support of a therapist.

#### 3.7.7. Nausea, Vomiting, Loss of Appetite, and Weight Loss

A differential diagnosis has to be carried out between TKI-related symptoms and disease progression, including the state of neoplastic cachexia.

Prevention: check patient’s weight, nutrition and physical activity during treatment. Coffee, alcohol, chocolate, and smoking could increase nausea [64]. Avoid eating favorite foods, and prefer small and varied meals.

AE practical advice: use a high-protein and high-calorie diet, nutritional supports, anti-emetic drugs and appetite stimulants as indicated by the specialist.

## 4. Discussion

Sorafenib, lenvatinib, vandetanib and cabozantinib have demonstrated effectiveness in the treatment of advanced, unresectable, refractory DTCs and MTCs [21,22,23,24]. However, the long term use and daily assumption of these drugs away from hospital settings, requires effective and proactive management of their common but rarely severe and life-threatening AEs, to keep patients on the treatment. AE prevention should be managed from the baseline, mainly through educational interventions and supportive care, to promote lifestyles changes and prevent poor adherence, dose reductions and discontinuation of therapy [65]. Patient education should include lifestyle improvement and methods of recognizing the signs and symptoms related to the onset of new toxicities early. For example, daily blood pressure self-assessment is more likely to detect a rise in blood pressure in outpatient follow-up [66]. Many mild AEs benefit from medical therapy and dose reduction, while in cases of severe toxicities, treatment can be temporarily interrupted or permanently discontinued [57,58]. Nevertheless, prevention remains an essential therapeutic approach. Therapy should be resumed at a lower dose after any interruptions to treatment, and after AE resolution [10]. As these compounds are cytostatic and non-cytotoxic [10,25], their action is not curative. They slow cancer progression, which resumes rapidly after discontinuation of therapy, therefore therapy interruption should be avoided, preferring a reduction in the daily dose [10]. As reported by Wiener et al. [39], even in thyroid cancer patients, more information is associated with a better perception of the disease, as well as with less distress and improved QoL. There are many factors associated with health-related quality of life (HRQoL) in patients age < 45, including hypocalcemia, post-operative dysphonia and dysphagia, presence of visible scarring and complications from radioactive iodine [67], especially for patients with persistent and RAI refractory disease [40]. According to Gallop et al. [40], some factors reduce the impact of disease on patient HRQoL: the right dosage of levothyroxine, coping styles, a good social support network, participation in psychological support groups, sharing the disease experience, competent health professionals who carefully consider the disease, not invalidating and diminishing it to being a “good cancer.” For this reason, in recent years thyroid cancers patients have been managed by a disease management team. This multidisciplinary healthcare practitioners group agrees on the therapeutic management and care of patients, with weekly meetings. In particular, the case manager of the team deals with the prevention and management of adverse events from TKI through a proactive approach, with the support of dedicated clinical pathways (as endocrinologic, surgery, dermatologic, etc.) as needed.

### 4.1. Relevance to Clinical Practice and Research

Nursing therapeutic education aimed towards the prevention, recognition and early treatment of adverse events related to the use of TKI, is essential to optimize therapeutic adherence and benefits for patients, reducing toxicities the negative impacts on QoL. The contribution of trained nurses and multidisciplinary teams is strongly recommended by the guidelines [14]. Due to their cytostatic action, it is important to avoid prolonged interruptions of therapy, and in the case of disease progression it is suggested to switch to another available treatment early on. A challenge for future research is the discovery of new molecules that improve the survival and management of thyroid cancer treatments alone or in combinations [27]. Future nursing research is needed to provide insight on this important topic.

### 4.2. Limitations

This study has some limitations. The review was conducted by a single researcher. Secondly—contrary to systematic reviews—narrative reviews are not based on standardized methodologies. Consequently, the results obtained and their generalizations should be carefully considered.

## 5. Conclusions

Thyroid cancer has an excellent prognosis in about 90% of cases, however, 10% of patients have advanced forms refractory to available treatments who can benefit from TKIs [21,22,23,24]. TKIs are not curative drugs, but they can induce disease stability, sometimes for a long time [2]. Although it is known that the disease will inevitably progress over time, it is important to keep patients on treatment for as long as possible, avoiding long and frequent interruptions, balancing the benefits and toxicities induced by the treatment. A specialist’s intervention before starting treatment and at the baseline is very important to address frequent TKI-related AEs, with a proactive approach aimed at the prevention and reduction of toxicities to keep the patient on therapy, increasing the benefits of the treatment. As widely indicated in cancer care, a multidisciplinary approach is strongly recommended in the diagnostic-clinical-therapeutic pathway of thyroid cancers, including the contribution of a senior nurse [38]. The contribution of a multidisciplinary healthcare practitioner team (HCPs) improves the management of TKIs [44], and places the basis of the essential therapeutic alliance [68], made up of sharing cognitive and emotional experiences, and promoting resilience in patients and the care team. The creation of a therapeutic alliance between the patient and the care team is essential to create a climate of trust and the sharing of objectives [69]. Through the use of the chronic care model, patients activated and involved in the choice of the most appropriate therapeutic pathway will show better satisfaction and adherence to the treatment [68]. Whenever possible, the presence of the caregiver is important from enrolment, as a special point of reference in the patient’s daily life and with respect to changes in their routine [45].

In our experience within an Italian Cancer Institute, the following members of the HCPs are present right from the beginning of treatment: an endocrinologist and an expert nurse accompanied by a dermatologist, an oncologist, a cardiologist, a psychologist, a dietician and pharmacist, available as needed during the clinical pathway. Nurses play a key role in the management of TKIs with their educational, proactive, risk reduction and benefit maximization functions. In their advocacy roles, they simplify the interaction between the patient and the care team, and are often the first and greatest interface between patients and their treatment-related needs [70]. In our experience, the support of a nurse Case Manager within the HCP team contributes to the planning of proactive and personalized assistance, the gold standard in the treatment of cancers and chronic diseases [71]. Printed information material, online sources, smartphone apps and the use of the teach-back method [72] can be useful to assess the information acquired by patients and caregivers. Mild AEs can initially be managed with symptomatic therapy and corrective educational nursing interventions (diet, lower sodium intake, physical activity, rest, proper skin cleansing and hydration, blood pressure measurement) [73] before dose-reduction, promoting treatment adherence. During outpatient follow-up visits, the nurse will be able to assess the onset of new symptoms or signs of infection, malnutrition, dehydration, cardio-circulatory changes, lack of self-care, depressive symptoms, asthenia and any changes in general conditions. Moreover, knowledge of the patient’s and family’s expectations of treatment and disease is important to support them in the acquisition of realistic and achievable goals at each stage of the therapeutic pathway.

In conclusion, adverse events during therapy with sorafenib, lenvatinib, cabozantinib and vandetanib are common to the four compounds, are frequently experienced by all patients at least once during treatment and are generally not life-threatening, excluding cardiovascular AEs [74]. A proactive educational nursing approach aimed at the prevention and correct management of toxicities during therapy, together with a contribution of a multidisciplinary healthcare team, is essential in optimizing therapeutic adherence, satisfying a patients’ need for information [39,75], maximizing benefits, reducing risks and medication errors [76], and positively impacting the QoL of both patient and their family.

## Figures and Tables

**Table 1:** Any-grade adverse events (adapted from [21,22,23,24]).

Drug	Tumour					Adverse Events (All Grades)						
		HFSR No (%)	Hypertension No (%)	Diarrhoea No (%)	Rash/Desquamation No (%)	Anorexia No (%)	Nausea No (%)	Wheight Loss No (%)	Mucositis No (%)	Fatigue No (%)	TSH Increase No (%)	QT Prolungation No (%)
Sorafenib	DTC	158 (76.3)	84 (40.6)	142 (68.6)	104 (50.2)	66 (31.9)	43 (20.8)	97 (46.9)	48 (23.2)	103 (49.8)	69 (33.3)	na
Lenvatinib	DTC	83 (31.8)	177 (67.8)	155 (59.4)	42 (16.1)	131 (50.2)	107 (41.0)	121 (46.4)	93 (35.6)	154 (59.0)	158 (61.5)	21 (8)
Cabozantinib	MTC	107 (50.0)	70 (32.7)	135 (63.1	41 (19.2)	98 (45.8)	92 (43.0)	102 (47.7)	62 (29.0)	87 (40.7)	125 (57)	na
Vandetanib	MTC	na	73 (32)	130 (56)	104 (45)	49 (21)	77 (33)	24 (10)	na	55 (24)	114 (49.3)	33 (14)

DTC: differentiated thyroid cancer; MTC: medullary thyroid cancer; No: number; HFSR: hand–foot skin reactions; TSH: thyroid-stimulating hormone; na: not applicable.
